# Exploring the Sound Absorption Potential of Ecoflex™ 00-35 for Soft and Flexible Noise Reduction

**DOI:** 10.3390/ma18194481

**Published:** 2025-09-25

**Authors:** Nourelhuda Mohamed, Manal Mohamed, Jae Gwan Kim

**Affiliations:** Biomedical Science and Engineering Department, Gwangju Institute of Science and Technology, Gwangju 61005, Republic of Korea; nonoalhodaali@gm.gist.ac.kr (N.M.); manalalnosh@gm.gist.ac.kr (M.M.)

**Keywords:** Ecoflex silicone rubbers, pressure, microstructure, sound absorption characteristics

## Abstract

This study investigates the acoustic performance of Ecoflex™ 00-35, a highly flexible silicone rubber, for use in soft and adaptable vibration and noise control systems. Under normal conditions, Ecoflex™ 00-35 consists of two components—Part A and Part B—which are mixed and cured at room temperature to form an elastomer. In this study, curing parameters such as the A/B mixing ratio, thinning agent addition, and curing pressure were varied to examine their effects on acoustic behavior. The microstructure of the prepared samples was analyzed using scanning electron microscopy (SEM), while sound absorption properties were measured using impedance tubes. Test results demonstrated that modifying curing parameters, applying vacuum, and incorporating a thinning agent increased the average cell diameter, leading to the fabrication of a moderate sound absorber with a sound absorption coefficient ranging from 0.35 to 0.60 in the low- to mid-frequency ranges. Further enhancement in low-frequency absorption was achieved by applying low pressure for a short duration, allowing cell expansion. In contrast, the addition of a thinning agent significantly improved absorption at higher frequencies. These findings highlight the influence of processing conditions on the acoustic behavior of soft silicone elastomers and provide valuable insights into their structure–property relationships. Ultimately, this study contributes to the development of advanced materials for acoustic damping and noise control applications.

## 1. Introduction

Sound absorption and noise reduction are essential considerations in various engineering applications, ranging from architectural acoustics to industrial noise control [1,2,3]. Most acoustic studies focus on traditional sound-absorbing materials such as foams, fibers, and composite structures to mitigate unwanted noise [4,5,6,7]. However, these materials often suffer from limitations, including brittleness, environmental degradation, and restricted adaptability to complex geometries [8,9,10]. To overcome these challenges, researchers have increasingly explored polymeric materials, particularly silicone rubbers, due to their unique viscoelastic properties, durability, and tunability for specific acoustic responses [11,12,13,14,15].

Among silicone-based materials, Ecoflex™ 00-35 is a soft and highly flexible platinum-cured elastomer known for its exceptional mechanical flexibility, low durometer hardness, and high elongation at break [16]. In addition, the low Young’s modulus and inherent viscoelasticity of Ecoflex™ 00-35 enable effective energy dissipation, making it well-suited for vibration damping and broadband noise control applications [17]. These properties make it a promising candidate for applications requiring soft, deformable, and resilient sound-absorbing materials. While extensive research has been conducted on the acoustic properties of various silicone rubbers [18,19,20,21,22], no studies have specifically investigated Ecoflex™ 00-35 for sound absorption or noise attenuation. The influence of factors such as porosity, filler incorporation, and structural modifications on the acoustic behavior of this material remains unexplored, presenting a significant gap in the current literature.

One effective method for enhancing the acoustic performance of silicone-based materials is the introduction of controlled porosity, which can be achieved using a vacuum oven [23,24]. The process involves subjecting the silicone mixture to a vacuum environment before or during the curing stage to create a porous microstructure. By adjusting vacuum pressure, curing time, and degassing cycles, it is possible to control the size and distribution of pores within the material, thereby improving its ability to trap and dissipate sound waves [25]. Studies have shown that porous silicone structures exhibit higher sound absorption coefficients, particularly at mid-to-low frequencies, due to the increased surface area and enhanced viscoelastic dissipation of acoustic energy [21,26,27,28]. Despite these promising findings, up to our knowledge, no previous research has been conducted on optimizing the vacuum-induced porosity of Ecoflex™ 00-35 specifically for soundproofing applications.

The primary objective of this study is to evaluate how curing parameters, vacuum-induced porosity, and the addition of a thinning agent affect the microstructure (pore size and distribution) and sound absorption performance of Ecoflex™ 00-35 silicone rubber for noise reduction applications. Samples were prepared by systematically varying the curing time, pressure, and thinning agent concentration. The resulting microstructure was characterized using SEM to investigate the effects of these elaboration parameters on pore morphology. Finally, the acoustic absorption coefficients of the different samples were measured using an impedance tube to assess their performance.

## 2. Materials and Methods

### 2.1. Materials

Ecoflex™ 00-35 Fast (Smooth-On, Inc., Macungie, PA, United States) is an ultra-soft, platinum-cured silicone elastomer designed for high flexibility, rapid curing, and skin-safe applications, making it ideal for prosthetics, wearable devices, and medical simulations. Its curing mechanism follows a hydrosilylation reaction (Equation (1)), where vinyl-functionalized silicone (Si-CH=CH_2_) reacts with hydride-functionalized silicone (Si-H) in the presence of a platinum catalyst, forming a cross-linked structure without releasing byproducts:

(1) $$\underset{\mathrm{Curing}\;\mathrm{agent}\;B}{\underset{︸}{-\overset{|}{\underset{|}{\mathrm{Si}}}-H}}+\underset{\mathrm{Polymer}\;A}{\underset{︸}{H2C=\mathrm{CH}-\overset{|}{\underset{|}{\mathrm{Si}}}-}}\overset{\mathrm{Catalyst}}{\underset{\mathrm{Pt}}{\to }}-\overset{|}{\underset{|}{\mathrm{Si}}}-\mathrm{CH}2-\mathrm{CH}2-\overset{|}{\underset{|}{\mathrm{Si}}}-$$

This reaction results in a dense, non-porous material with negligible shrinkage and excellent biocompatibility [29,30]. Compared to Ecoflex™ 00-30, the 00-35 Fast version has a significantly shorter pot life (~2.5 min) and faster cure time (~10 min), making it suitable for rapid prototyping and high-efficiency production. While it provides moderate vibration damping, its low inherent porosity limits sound absorption compared to open-cell foams. Meanwhile, porosity can be controlled using various methods: mechanical aeration introduces microbubbles, expandable microspheres create controlled voids, solvent leaching removes a dispersed phase to leave pores, and vacuum degassing before curing eliminates trapped air to maintain a smooth, bubble-free structure. These techniques allow for tailored porosity, enhancing acoustic properties for specialized medical and noise-reducing applications.

### 2.2. Experimental Protocol

Different samples were prepared by systematically varying three curing parameters: (i) the A/B mixing ratio, (ii) the addition of a thinning agent (silicone oil), and (iii) the curing pressure and time inside a vacuum oven (DAEYANG ETS, Hwaseong-si, South Korea) in order to examine their effects on the microstructure and acoustic properties of silicone rubber. Each sample was fabricated with a diameter of 100 mm and a thickness of 15 mm to ensure consistency in testing.

#### 2.2.1. A/B Ratio

According to the manufacturer, a 1:1 ratio of Part A to Part B is recommended for preparing the silicone rubber. However, in this study, the A/B ratio was varied to explore its impact on the material’s microstructure and acoustic properties.

#### 2.2.2. Thinning Agent

We used KF-96 silicone oil (Shin-Etsu Chemical Co., Ltd., Tokyo, Japan), a synthetic oil with a dimethyl polysiloxane structure that does not occur naturally. Composed of heat-resistant siloxane bonds (Si-O-Si) and organic methyl groups, it offers unique properties compared to conventional mineral and synthetic oils. Due to its thermal stability, chemical resistance, and versatility, KF-96 is widely used in industries such as electronics, transportation, chemicals, office equipment, cosmetics, and textiles. In this study, we added 25% KF-96 silicone oil as a thinning agent, based on the reference sample, to investigate its effect on the material’s properties.

#### 2.2.3. Pressure

**Figure 1:**
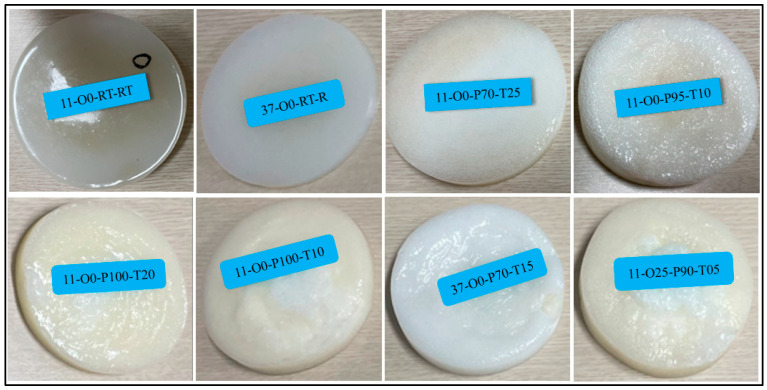
Photographs of the prepared samples after curing and demolding.

Different pressure values were applied to the samples during the curing process to investigate their impact on the microstructure and acoustic behavior of the foams. These pressures were applied for various time intervals inside a vacuum oven, which creates a sub-atmospheric pressure environment within the chamber. The vacuum process helps control the cellular structure of the material by promoting the formation of pores, which in turn affects its acoustic properties. By varying both the pressure values and curing times, this experiment aimed to explore how these factors influence the material’s porosity and its ability to absorb sound. Table 1 provides the sample codes, formulations, and processing conditions of the prepared Ecoflex™ materials, and Figure 1 shows photographs of them.

### 2.3. Measurement Methods

Understanding the relationship between a material’s microstructure and its macroscopic properties is crucial for predicting its functional performance. This knowledge enables the optimization of the material for specific practical applications. In this study, scanning electron microscopy (SEM) analysis was used to examine the microstructure, while the sound absorption coefficient was measured to evaluate the material’s acoustic properties. Also, the noise reduction coefficient (NRC) and sound absorption average (SAA) were computed as standardized single-number indicators of each material’s sound absorption performance.

#### 2.3.1. Microgeometry Analysis

The average pore diameter and distribution were determined using SEM. SEM imaging was performed using a Verios 5 scanning electron microscope (Thermo Fisher Scientific, Waltham, MA, USA) in secondary electron mode with an Everhart–Thornley detector. Images were acquired at an accelerating voltage of 5.00 kV, beam current of 0.10 nA, and a working distance of 6.2 mm. Magnifications is 100×, corresponding to horizontal field widths of 2.07 mm. Samples were mounted on metal stubs using adhesive tape and coated with platinum for conductivity. Image analysis was conducted using ImageJ software (ImageJ 1.52a.), and the average cell diameter was calculated by fitting the pore distribution to a normal distribution.

#### 2.3.2. Absorption Coefficient and Surface Impedance

The sound absorption coefficient and surface impedance were measured using a two-microphone impedance tube (SCIEN model no. 9301) (ORTEC, Oak Ridge, TN, USA), following the standard procedure outlined in KS F 2814-2: 2022 [31]. The tests were conducted on cylindrical rubber samples with a 100 mm diameter and 15 mm thickness across a frequency range of 125–1800 Hz. The two-microphone transfer-function method was applied, with calibration performed prior to each measurement. Tests were conducted at a temperature of 25 ± 3 °C, relative humidity of 51 ± 5%RH, and atmospheric pressure of 999.6 ± 3 hPa. The incident sound wave was directed normally to the surface of the sample. While the absorption coefficient and surface impedance are primarily influenced by sample thickness, slight variations in thickness occurred due to the different pressures applied during curing, which sometimes caused the samples to expand and become thicker. These variations in thickness were considered during the analysis.

#### 2.3.3. Calculation of NRC and SAA

The noise reduction coefficient (NRC) and sound absorption average (SAA) were calculated to provide standardized single-number ratings of the materials’ sound absorption performance. NRC was determined as the arithmetic average of the sound absorption coefficients at 250, 500, 1000, and 1600 Hz (the nearest available measurement to 2000 Hz), rounded to the nearest 0.05. SAA was calculated as the average of the absorption coefficients across the available 1/3-octave band center frequencies from 200 to 1600 Hz, rounded to the nearest 0.01, in accordance with ASTM C423 [32].

## 3. Results and Discussion

### 3.1. Microstructure Characteristics

**Figure 2:**
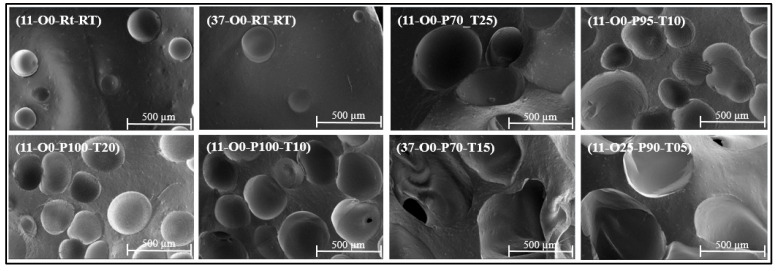
SEM images of the prepared samples.

The acoustic properties of each material are closely linked to the microstructure of the samples. Figure 2 presents the SEM image of the prepared silicone rubber samples. Upon examining the SEM image of the reference sample (11-O0-RT-RT), it is evident that the sample has a low pore count, which can be attributed to the low-viscosity, platinum-cure (addition-cure) silicone formulation. This formulation is designed to produce a dense, uniform elastomer rather than a porous structure. Even when the A/B ratio was altered (sample 37-O0-RT-RT), the resulting sample still exhibited a low number of pores with small diameters. Table 2 summarizes the calculated average cell diameter for the prepared samples.

For sample 11-O0-P70-T25, the average cell diameter is 0.72 mm, significantly larger than that of the 11-O0-RT-RT reference sample, which has an average diameter of 0.26 mm. This increase in cell size can be attributed to the application of high pressure during the curing process, which causes trapped air bubbles to expand, resulting in a more porous structure and larger cell diameter. For samples 11-O0-P95-T10, 11-O0-P100-T20, and 11-O0-P100-T10, the cell diameter is a bit similar, and larger than that of 11-O0-RT-RT, due to the small difference in the 5 kPa pressure applied during curing. The increase in cell diameter results from the pressure causing the cells to expand; however, during vacuum evacuation, this expansion collapses, limiting further enlargement. Notably, the average cell diameter of 11-O0-P95-T10, 11-O0-P100-T20, and 11-O0-P100-T10 is smaller than that of 11-O0-P70-T25, as the pressure was lower and applied for a shorter duration, preventing the pores from maintaining their expanded size. Among all the prepared samples, 37-O0-P70-T15 has the largest cell diameter of 0.93 mm. This sample was prepared by adjusting both the A/B ratio and curing parameters. The high pressure and extended curing time allowed the pores to expand significantly, and they were able to retain their size during vacuum evacuation as the material reached the stabilization and full cure stage. It is worth noting that, despite spending less time in the oven and having the same pressure as the 11-O0-P70-T25 sample, this sample has a larger cell diameter. This could be due to the change in the A/B ratio, which can influence the viscosity and curing kinetics of the silicone, potentially affecting the formation of pores and the overall cellular structure. Further investigation is required to fully understand the combined effects of these factors. For sample 11-O25-P90-T05, the average cell diameter was found to be 0.85 mm, significantly larger than the reference sample. This increase is primarily due to the addition of silicone oil, which reduces the overall viscosity of the mixture, allowing trapped air bubbles to expand more freely before the onset of visible gelation.

Therefore, material porosity can be effectively controlled by adjusting the curing pressure and duration inside the vacuum oven. Larger pores can be obtained by applying higher pressure for a longer time, while smaller pores can be achieved by using lower pressure for a shorter duration. Furthermore, the addition of silicone oil helps stabilize the large pores that form, even when pressure is applied for shorter durations.

### 3.2. Acoustic Behavior

The acoustic performance of the prepared samples was analyzed, with the samples categorized based on their preparation parameters. The following subsections will discuss the impact of various curing parameters.

#### 3.2.1. Effect of Pressure Variation at Fixed Curing Time

**Figure 3:**
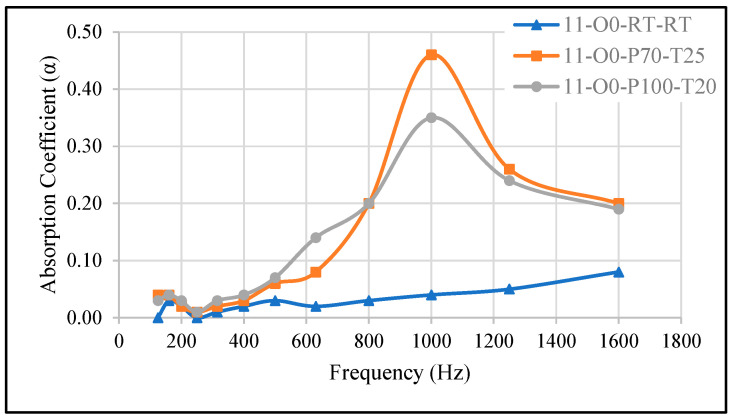
Effect of pressure variation on the SAC. 11-O0-RT-RT: reference sample, 11-O0-P70-T25: sample cured in the oven at −70 kPa, 11-O0-P100-T20: sample cured in the oven at −100 kPa.

Figure 3 illustrates the sound absorption coefficient (SAC) of samples 11-O0-RT-RT, 11-O0-P70-T25, and 11-O0-P100-T20. The reference sample (11-O0-RT-RT) exhibits a very low SAC across the measured frequency range. However, when the same sample was cured inside the vacuum oven at −70 kPa (11-O0-P70-T25) and −100 kPa (11-O0-P100-T20) for 20 to 25 min, the SAC increased, reaching a peak of 0.46 for 11-O0-P70-T25 and 0.35 for 11-O0-P100-T20, both at 1000 Hz. This enhancement in SAC can be attributed to the formation of pores during the curing process, which significantly influences the material’s acoustic behavior.

The differences between 11-O0-P70-T25 and 11-O0-P100-T20 can be explained by the absorption at mid frequencies, leading to a higher SAC peak. Conversely, at −100 kPa (lower pressure, higher vacuum), the pores expand more freely, creating a more open and interconnected structure, which broadens the absorption curve and improves low- effect of pressure on pore structure. At −70 kPa (relatively higher pressure, lower vacuum), the trapped air bubbles expand moderately, forming a structure that enhances sound frequency sound absorption. This results in SAC starting to increase at lower frequencies (600 Hz), although the peak absorption remains slightly lower than that of 11-O0-P70-T25.

These results highlight the importance of curing pressure in tailoring porosity, as higher pressures promote localized sound absorption, while lower pressures generate a broader absorption profile by facilitating pore expansion and interconnectivity.

#### 3.2.2. Effect of Time Variation at Fixed Curing Pressure

**Figure 4:**
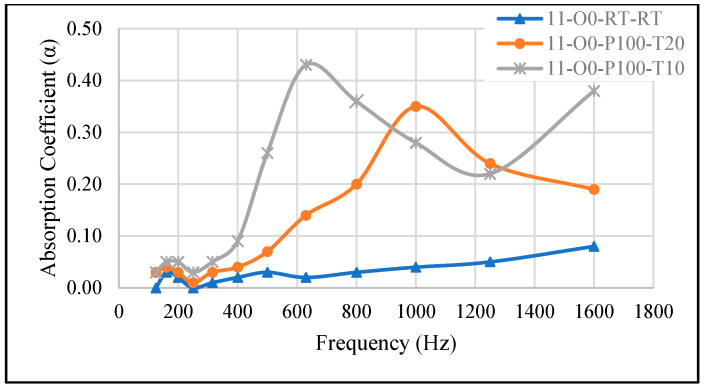
Effect of curing time in the oven on the SAC. 11-O0-RT-RT: reference sample, 11-O0-P100-T20: cured for 20 min, 11-O0-P100-T10: cured for 10 min.

Figure 4 illustrates the effect of curing time on the SAC while maintaining a constant pressure of −100 kPa. When the reference sample (11-O0-RT-RT) was cured for 10 min (11-O0-P100-T10) and 20 min (11-O0-P100-T20), the SAC increased, reaching 0.43 for 11-O0-P100-T10 and 0.35 for 11-O0-P100-T20. Additionally, the peak absorption frequency shifted, occurring at 1000 Hz for 11-O0-P100-T20 and 630 Hz for 11-O0-P100-T10.

These differences can be explained by the evolution of pore structure over time. In 11-O0-P100-T10 (10 min curing time), the material had less time to stabilize, allowing the expanded air pockets to remain relatively large and more interconnected. Larger pores interact more effectively with low-frequency sound waves, resulting in a higher SAC at lower frequencies (630 Hz).

On the other hand, in 11-O0-P100-T20 (20 min curing time), the extended curing duration allowed the material to further stabilize, leading to smaller, more evenly distributed pores. Smaller pores enhance mid-frequency absorption, shifting the SAC peak to 1000 Hz while slightly reducing overall absorption compared to 11-O0-P100-T10. These findings further validate the results presented in Section 3.2.1.

Thus, shorter curing times promote low-frequency absorption by maintaining larger pores, whereas longer curing times favor higher-frequency absorption due to the formation of a denser microstructure with smaller pores. This demonstrates that curing time can be adjusted to optimize absorption performance across different frequency ranges.

#### 3.2.3. Effect of Minor Pressure Changes and Shorter Curing Time

**Figure 5:**
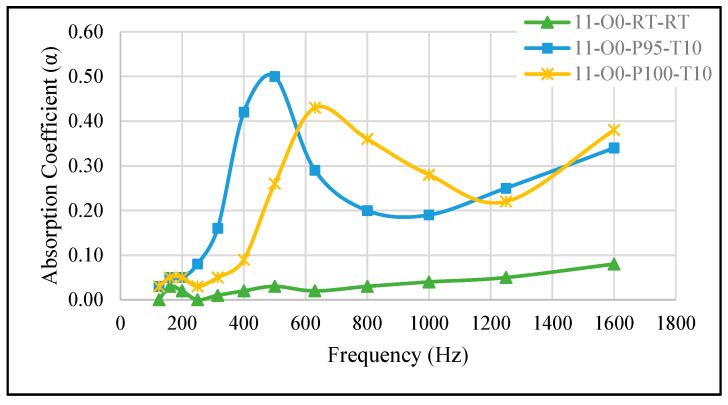
Effect of slight pressure variation and shorter curing time on the SAC. 11-O0-RT-RT: reference sample, 11-O0-P95-T10: cured at −95 kPa, 11-O0-P100-T10: cured at −100 kPa.

When the samples were cured inside the vacuum oven at pressures of −95 kPa (11-O0-P95-T10) and −100 kPa (11-O0-P100-T10) for 10 min, their SAC increased compared to the reference sample (11-O0-RT-RT), with well-defined maxima of 0.50 and 0.43, respectively. Figure 5 presents the SAC curves of these samples. Notably, sample 11-O0-P95-T10 exhibits a higher SAC than 11-O0-P100-T10, with its peak occurring at a slightly lower frequency. Meanwhile, sample 11-O0-P100-T10 demonstrates moderate SAC values over a broader frequency range. This trend can be attributed to the influence of vacuum pressure on the material’s porosity. The lower vacuum (−95 kPa) in sample 11-O0-P95-T10 likely resulted in larger, more irregular pores (see Figure 1), which are more effective at absorbing lower-frequency sounds due to their interaction with longer wavelengths. Conversely, the higher vacuum (−100 kPa) in sample 11-O0-P100-T10 facilitated more uniform but smaller pores, leading to a broader SAC response but with a slightly lower peak. These findings align with previous observations, confirming that adjusting the vacuum level during curing can effectively tailor the sound absorption properties of the material. Additionally, combining higher pressure with shorter curing time can further enhance SAC at lower frequencies, as the rapid expansion of trapped air during curing promotes the formation of larger and irregular pores, which are particularly effective in attenuating low-frequency sound waves. Please note that although the average cell diameter of sample 11-O0-P95-T10 was found to be smaller than that of sample 11-O0-P100-T10 (see Table 2), the irregularity in pore distribution played a significant role in shifting the SAC to lower frequencies.

#### 3.2.4. Combined Effect A/B Ratio and Curing Pressure

**Figure 6:**
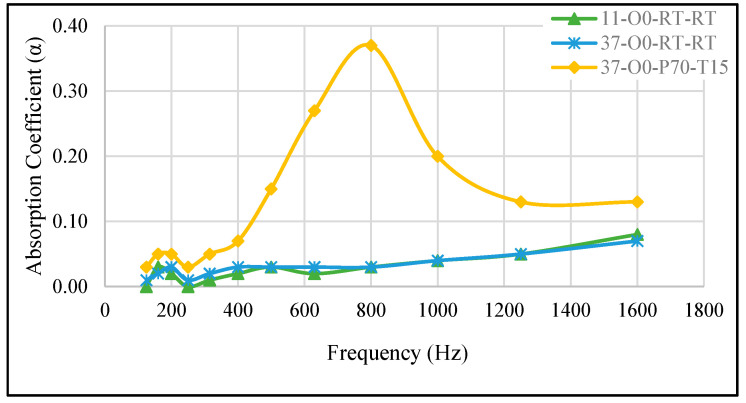
The effect of changing the A/B mixing ratio on the SAC: 11-O0-RT-RT: reference sample (A/B = 1), 37-O0-RT-RT: A/B = 30/70, 37-O0-P70-T15: A/B = 30/70 and cured at −70 kPa for 15 min.

Figure 6 illustrates that altering the ratio between parts A and B in the preparation of the sample resulted in minimal changes in the SAC between samples 11-O0-RT-RT and 37-O0-RT-RT. However, when the A/B ratio was adjusted and the sample was cured under a vacuum at −70 kPa for 15 min (37-O0-P70-T15), the SAC increased, reaching a peak of 0.37 at 800 Hz. Notably, the frequency range for this sample (37-O0-P70-T15) falls between the lower and higher frequencies observed for samples prepared with an A/B ratio of 1, and the peak SAC (0.37) is lower than that of the sample with the same curing pressure but an A/B ratio of 1 (sample 11-O0-P70-T25). This suggests that while the higher pressure enhanced the SAC, the combination of a different A/B ratio and curing conditions affected the material’s acoustic behavior. Further investigation is required to fully understand the effect of varying the A/B mixing ratio on the material’s sound absorption performance.

#### 3.2.5. Effect of Thinning Agent on Acoustic Performance

**Figure 7:**
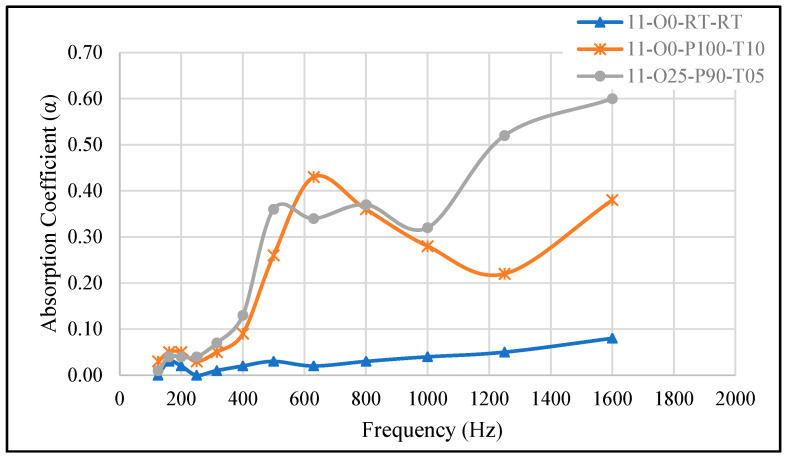
The effect of adding a thinning agent (silicone oil) on the SAC, 11-O0-RT-RT: reference sample, 11-O0-P100-T10: cured for 10 min at −100 kPa without oil, 11-O25-P90-T05: cured for 5 min at −90 kPa with 25% silicone oil.

In this experiment, we investigated the effect of adding a thinning agent (silicone oil) to the mixture to assess its impact on the sound absorption coefficient (SAC). A silicone oil was added at 25% of the total weight of the sample to prepare sample 11-O25-P90-T05. Figure 7 shows the performance of this sample compared to the reference (11-O0-RT-RT) and sample 11-O0-P100-T10, which was prepared under similar conditions but without the addition of oil. The results reveal that the addition of silicone oil has a similar effect on the SAC in low frequencies, with a broader absorption curve for the 11-O25-P90-T05 sample. Moreover, the presence of silicone oil resulted in the highest SAC at higher frequencies (SAC = 0.60 at 1600 Hz), and the SAC of sample 11-O25-P90-T05 spans a wider frequency range, starting from 500 Hz. The increase in SAC at higher frequencies can be attributed to the oil’s role in reducing the viscosity of the mixture, allowing for a more uniform distribution of pores. This uniformity improves the material’s ability to absorb sound energy across a broader frequency spectrum, particularly enhancing high-frequency absorption. Enhancing the SAC in high-frequency ranges through the addition of a thinning agent has already been reported in the literature [33].

In this study, silicone oil was incorporated only in sample 11-O25-P90-T05 as a targeted feasibility test to assess its potential to enhance sound absorption. The curing conditions for 11-O25-P90-T05 (–90 kPa, 5 min, A/B = 1:1) were selected based on the performance of sample 11-O0-P100-T10 (–100 kPa, 10 min, no oil), which had demonstrated broadband absorption characteristics without oil. This choice allowed us to directly evaluate the effect of oil addition under a baseline condition that was already acoustically favorable. The primary aim was to determine whether the addition of 25% silicone oil could broaden the absorption range and enhance high-frequency SAC, as reported in prior studies [33]. Due to scope and resource constraints, a full factorial study of oil content across multiple curing pressures and times was not performed in this work. Future research will systematically explore oil incorporation under different processing conditions to optimize both low- and high-frequency absorption performance.

Overall, the curing conditions were found to significantly influence the microstructure of the silicone foams, which in turn affects their sound absorption behavior across different frequency ranges. Specifically, higher curing temperatures or longer curing times led to larger, more open cells, promoting enhanced absorption at lower frequencies (125–500 Hz). In contrast, samples with smaller, denser cells—resulting from shorter curing times or lower temperatures—showed improved absorption at higher frequencies (1000–1800 Hz). This clear link between cell structure and frequency-dependent absorption aligns with the impedance tube measurements and highlights the importance of controlling curing parameters to tailor acoustic performance for specific noise environments.

In addition, the experiments revealed that varying curing parameters, pressure, and the addition of silicone oil significantly influenced the SAC of the silicone rubber samples. Table 3 provides a summary of the sample preparation conditions and the corresponding peak SAC values. The reference sample (11-O0-RT-RT) showed the lowest SAC across all frequencies. Increasing the curing pressure, as seen in sample 11-O0-P70-T25 (cured at −70 kPa) and 11-O0-P95-T10 (cured at −95 kPa), led to a noticeable increase in SAC, with sample 11-O0-P70-T25 achieving a maximum SAC of 0.46 at 1000 Hz. Similarly, increasing the curing time at higher pressure (sample 11-O0-P100-T10) also improved SAC, although the peak frequency shifted. The addition of silicone oil in sample 11-O25-P90-T05 resulted in the highest SAC of 0.60 at 1600 Hz, especially in higher frequencies, while also broadening the absorption range. Furthermore, adjusting the A/B ratio did not significantly affect the SAC, but altering the ratio while curing at high pressure (sample 37-O0-P70-T15) showed a moderate increase in SAC. These results indicate that pressure, curing time, and the incorporation of thinning agents have a considerable impact on the acoustic properties of the material, particularly at higher frequencies, with larger and more uniform pores contributing to enhanced sound absorption performance.

To place our results in context, Table 4 summarizes the peak SAC values and corresponding frequencies for all prepared samples in this study alongside representative values reported in the literature for silicone-based acoustic foams and composites. Our best-performing sample (11-O25-P90-T05) achieved a SAC of 0.60 at 1600 Hz, which is comparable to the performance of open-cell silicone foams reported by Abbad et al. [33], where SAC values of 0.55–0.65 were observed in the 1500–2000 Hz range for similar sample thicknesses. Peng et al. [19] achieved a peak SAC of ~0.58 at 1400 Hz for NaCl-templated silicone foams of comparable density. Huang et al. [18] demonstrated tunable absorption by incorporating mesoporous silica into silicone rubber, reaching SAC values up to ~0.62 at mid-to-high frequencies. These comparisons indicate that the modified Ecoflex™ 00-35 developed here performs on par with other silicone-based porous absorbers, while offering additional advantages in mechanical flexibility and process tunability.

### 3.3. Quantitative Acoustic Performance Assessment

Single-number ratings (NRC and SAA) were computed for all prepared samples to facilitate direct comparison of their broadband acoustic performance (Table 5). The results show that sample 11-O25-P90-T05 achieved the highest NRC (0.33) and SAA (0.28), indicating strong performance across the tested frequency range. This is consistent with its high measured SAC at both mid and high frequencies. Sample 11-O0-P95-T10 exhibited the second-highest NRC (0.28) and SAA (0.25), reflecting its enhanced absorption in the mid-frequency range. In contrast, samples 11-O0-RT-RT and 37-O0-RT-RT showed the lowest single-number ratings, confirming their limited acoustic effectiveness. These values allow for a direct comparison with commercial sound-absorbing materials and demonstrate the potential of tailoring curing conditions and composition to improve broadband performance.

## 4. Strength, Limitations, and Future Work

To the best of our knowledge, this work is the first to investigate the sound absorption performance of Ecoflex™ 00-35 under controlled porosity achieved through vacuum-assisted curing. While previous studies have explored the acoustic behavior of other silicone rubbers, no prior research has examined how varying curing pressure, time, and the incorporation of thinning agents can tune the microstructure and frequency-dependent absorption of this specific ultra-soft elastomer. This novelty not only addresses a gap in the literature but also expands the potential applications of Ecoflex™ 00-35 beyond its conventional use in biomedical and wearable devices, positioning it as a viable candidate for adaptable, soft, and durable noise control systems. The inclusion of literature comparisons further validates the material’s competitive performance compared to other silicone-based absorbers.

Of note, this study was designed as an exploratory investigation to identify key processing–structure–property relationships for Ecoflex™ 00-35 rather than to conduct a full-factorial assessment of all possible combinations of pressure and curing time. While the selected conditions captured the most relevant variations in pore morphology and sound absorption, future work will implement a full-factorial study to systematically refine and validate the observed processing–acoustic relationships, thereby providing stronger support for the conclusions drawn here.

Given the exploratory scope of this work, certain limitations remain. For instance, the moderate sound absorption achieved (with a coefficient of 0.35–0.60 in the low- to mid-frequency range) may not be sufficient for high-performance noise reduction applications. Additionally, the study focused primarily on low- to mid-frequency absorption, leaving very low-frequency (<125 Hz) performance relatively unexplored.

To overcome these limitations, Ecoflex™ 00-35 could be integrated into composite or multilayer systems. For example, coupling the porous Ecoflex™ layer with a micro-perforated or perforated facing backed by a tuned air cavity can enhance low-frequency attenuation through quarter-wavelength resonance, while maintaining mid- to high-frequency dissipation. Incorporating Helmholtz or membrane resonators either behind or within the Ecoflex™ layer can supply targeted low-frequency peaks, and graded-porosity laminates—fabricated by stacking layers produced under different curing conditions—can improve impedance matching across a wide frequency range. In addition, thin resistive skins (e.g., nonwovens or micro-textured films) may be applied to increase high-frequency viscous losses. These design adaptations, which are well-established in porous–resonant hybrid systems [21,26,27,34,35] could enable Ecoflex™-based absorbers to achieve stronger, broader absorption while retaining the mechanical compliance, durability, and conformability that make them attractive for wearable, biomedical, and adaptive acoustic applications.

The vacuum-induced porosity method employed in this study provides only partial control over pore size distribution and interconnectivity, which are critical for optimizing acoustic performance. More precise tailoring of pore structures could be achieved using alternative approaches such as foaming agents, salt leaching, or gas expansion techniques. Foaming agents can generate uniform bubbles within the polymer matrix, enabling controlled porosity and pore size [36,37]. Salt leaching involves incorporating soluble particles that can later be removed to produce interconnected pores with predictable dimensions [38,39]. Gas expansion methods, such as CO_2_ or nitrogen foaming, allow the regulation of pore size and connectivity through pressure and temperature adjustments [40]. Incorporating these approaches in future work could enable the systematic design of pore architectures, optimizing both the absorption coefficient and the frequency range of acoustic performance.

Ecoflex™ 00-35 demonstrates excellent chemical stability; however, its long-term durability under practical conditions, including mechanical wear, UV exposure, and elevated temperatures, may be limited. Even if not tested here, potential limitations can be mitigated through strategies such as incorporating UV stabilizers [41], reinforcing fillers [42], or protective coatings to enhance mechanical robustness and environmental resistance. Future studies should systematically evaluate these approaches to ensure sustained performance in real-world applications.

Finally, while the primary focus of this study was the acoustic performance of Ecoflex™ 00-35 under various curing conditions, the mechanical properties of the prepared samples are also crucial for flexible noise-control applications. According to the manufacturer, unmodified Ecoflex™ 00-35 exhibits a tensile strength of ~1.38 MPa, elongation at break of ~900%, and a Young’s modulus of 69–138 kPa [16], consistent with previously reported data for platinum-cured silicone elastomers [33]. Modifications in this work, including vacuum curing, variation in the A/B ratio, and silicone oil addition, may influence these properties by altering crosslink density and microstructural porosity. While direct tensile testing was not performed here, previous studies on modified silicone systems suggest that mechanical flexibility is largely retained [43]. Future work will include systematic stress–strain measurements to quantify these effects and ensure that the materials maintain the flexibility required for wearable, biomedical, and adaptive acoustic applications.

## 5. Conclusions

This study explores the potential of Ecoflex™ 00-35, a highly flexible silicone rubber, for vibration and noise control applications, addressing the need for soft, durable, and adaptable acoustic materials. Unlike traditional sound absorbers such as fiberglass and polyurethane foams, which can be brittle and environmentally unstable, Ecoflex™ 00-35 offers superior elasticity, chemical stability, and biocompatibility, making it ideal for wearable devices, automotive noise reduction, and adaptive acoustic panels. By modifying curing parameters and applying vacuum, a moderate sound absorber with a sound absorption coefficient of 0.35 to 0.60 in the low- to mid-frequency range was fabricated. The calculated NRC and SAA values confirm that the optimized samples, particularly 11-O25-P90-T05 and 11-O0-P95-T10, achieve competitive broadband absorption performance, supporting the feasibility of tuning Ecoflex™-based foams for targeted noise control applications. The findings of this study have direct implications for the design and implementation of noise control materials in real-world applications. By demonstrating how curing conditions influence cell structure and frequency-dependent sound absorption, our results provide a pathway to tailor silicone-based foams for specific acoustic environments. Due to its unique combination of high elasticity, durability, and biocompatibility, it is particularly suitable for applications where precise acoustic performance and material reliability are essential. Such applications include medical devices, wearable sensors, specialized acoustic panels, and soft robotics, where cheaper alternatives may compromise performance or longevity.

## Figures and Tables

**Table 1:** Sample codes, formulations, and processing conditions of the prepared Ecoflex™ materials.

Sample	%A	%B	%Oil	Curing Temp.	Pressure (kPa)	Time in Vacuum (min)
11-O0-RT-RT(ref)	50	50	-	Room	-	-
11-O0-P70-T25	50	50	-	-	–70	25
11-O0-P100-T20	50	50	-	-	–100	20
11-O0-P95-T10	50	50	-	-	–95	10
11-O0-P100-T10	50	50	-	-	–100	10
37-O0-RT-RT	30	70	-	Room	-	-
37-O0-P70-T15	30	70	-	-	–70	15
11-O25-P90-T05	50	50	25	-	–90	5

**Table 2:** Average cell diameter of the prepared samples.

Sample	11-O0-RT-RT	37-O0-RT-RT	11-O0-P70-T25	11-O0-P95-T10	11-O0-P100-T20	11-O0-P100-T10	37-O0-P70-T15	11-O25-P90-T05
Average cell diameter (mm)	0.26	0.26	0.72	0.35	0.38	0.46	0.93	0.85
SD cell diameter (mm)	0.06	0.11	0.15	0.08	0.08	0.08	0.13	0.25

**Table 3:** A summary table of sample preparation conditions and corresponding peak SAC values.

Sample	Description	Peak SAC	Peak Freq. (Hz)
11-O0-RT-RT	A/B = 1:1, no oil, cured at room temperature, no vacuum	0.07	1600
11-O0-P70-T25	A/B = 1:1, no oil, cured at –70 kPa for 25 min	0.46	1000
11-O0-P100-T20	A/B = 1:1, no oil, cured at –100 kPa for 20 min	0.35	1000
11-O0-P95-T10	A/B = 1:1, no oil, cured at –95 kPa for 10 min	0.50	500
11-O0-P100-T10	A/B = 1:1, no oil, cured at –100 kPa for 10 min	0.43	630
37-O0-RT-RT	A/B = 3:7, no oil, cured at room temperature, no vacuum	0.08	1600
37-O0-P70-T15	A/B = 3:7, no oil, cured at –70 kPa for 15 min	0.37	800
11-O25-P90-T05	A/B = 1:1, 25% silicone oil, cured at –90 kPa for 5 min	0.60	1600

**Table 4:** Peak sound absorption coefficient (SAC) values of prepared Ecoflex™ samples compared with literature-reported silicone-based absorbers.

This Study Performance	Comparable Literature Performance	Reference
11-O0-RT-RT, SAC = 0.08 at 1.6 kHz	Dense unmodified silicone rubber, SAC < 0.15	[33]
11-O0-P70-T25, SAC = 0.46 at 1 kHz	Open-cell silicone foam, SAC 0.45–0.55 at 1–1.5 kHz	[33]
11-O0-P100-T20, SAC = 0.35 at 1 kHz	NaCl-templated silicone foam, SAC~0.35–0.40 at 1–1.2 kHz	[19]
11-O0-P95-T10, SAC = 0.50 at 500 Hz	Porous silicone with irregular pores, SAC 0.48–0.53 at 800–1000 Hz	[33]
11-O0-P100-T10, SAC = 0.43 at 630 Hz	Large-pore silicone foam, SAC~0.40–0.45 at 600–800 Hz	[19]
37-O0-RT-RT, SAC = 0.07 at 1.6 kHz	Low-porosity silicone, SAC < 0.15	[33]
37-O0-P70-T15, SAC = 0.37 at 800 Hz	High-pressure cured silicone foam, SAC~0.35–0.40 at 800 Hz	[19]
11-O25-P90-T05, SAC = 0.60 at 1.6 kHz	Mesoporous silica–silicone composite, SAC 0.58–0.62 at 1.4–1.6 kHz	[18]

**Table 5:** NRC and SAA of the prepared samples.

Sample	NRC	SAA
11-O0-RT-RT	0.04	0.03
11-O0-P70-T25	0.18	0.13
11-O0-P100-T20	0.15	0.13
11-O0-P95-T10	0.28	0.25
11-O0-P100-T10	0.24	0.21
37-O0-RT-RT	0.04	0.03
37-O0-P70-T15	0.13	0.14
11-O25-P90-T05	0.33	0.28

## Data Availability

The original contributions presented in this study are included in the article. Further inquiries can be directed to the corresponding author.

## Abbreviations

The following abbreviations are used in this manuscript:

SEM	Scanning Electron Microscopy
SAC	Sound Absorption Coefficient
NRC	Noise Reduction Coefficient
SAA	Sound Absorption Average
